# Extramedullary Plasmacytoma of the Nasal Cavity Report of Three Cases With Review of the Literature

**DOI:** 10.5812/ircmj.2209

**Published:** 2013-04-05

**Authors:** Mohamad Javad Ashraf, Negar Azarpira, Bighan Khademi, Elham Abedi, Afsoon Hakimzadeh, Bita Valibeigi

**Affiliations:** 1 Pathology Department, Shiraz University of Medical Sciences, Shiraz, IR Iran; 2 Transplant Research Center, Shiraz University of Medical Sciences, Shiraz, IR Iran; 3 Otolaryngology Department, Shiraz University of Medical Sciences, Shiraz, IR Iran

**Keywords:** Plasmacytoma, Sinonasal Undifferentiated Carcinoma, Antigens, CD38

## Abstract

Extramedullary plasmacytoma is a rare neoplasm characterized by monoclonal proliferation of plasma cells. Most lesions occur in the head and neck, primarily in the upper aerodigestive tract. The nasal cavity and nasal septum are the most common sites of occurrence. In this report, three patients admitted in our clinic with history of nasal obstruction and/or epistaxis. Patients were diagnosed with extramedullary plasmacytoma and mass were completely excised. This entity usually occurred in 5th-6th decade of life. One of our patients, a young man, was completely asymptomatic and following a paroxysm of coughing, a polypoid mass was expectorated. The clinical and histopathologic findings of plasmacytoma are discussed. In order to exclude systemic involvement, systematic approach using clinical, laboratory, and radiologic investigations was performed. Extramedullary plasmacytoma of the nasal cavity is rare and should be considered in the differential diagnosis of nasal cavity masses especially in young age group.

## 1. Introduction

Plasma cell neoplasms (plasma cell dyscrasias) are characterized by neoplastic proliferation of a single clone of plasma cells, producing a monoclonal immunoglobulin. Plasma cell neoplasms can present as a single lesion (solitary plasmacytoma) or as multiple lesions (multiple myeloma). Solitary plasmacytomas present as solitary plasmacytoma of bone (SPB), and extramedullary plasmacytoma (EMP) (1, 2). EMP are most often located in the head and neck region, commonly affecting the nasal cavity, paranasal sinuses, tonsillar fossa, and oral cavity but may also occur in the gastrointestinal tract, urinary bladder, gland, lymph nodes, and skin (3-5). In nasal cavity, they represent approximately 4% of tumors. It accounts for approximately 1 – 3% of human malignancies, with median age of 55 to 60 years, and approximately two-thirds of patients are male (3, 4). The etiology of this disease remains unknown, but chronic irritation from inhaled irritants and viral pathogenesis has been suggested (2). Most patients present with symptoms such as epistaxis, nasal discharge (rhinorrhea) or nasal obstruction (3, 4). It is important to distinguish EP from other plasma cell tumors for the purposes of prognosis and treatment. The main differential diagnosis is multiple myeloma and Waldenstrom macroglobulinemia. The evaluation of a patient with a suspected EP should include a biopsy of the suspected lesion for tissue histological confirmation, a unilateral bone marrow aspirate and biopsy, and laboratory studies. Imaging should include a metastatic bone survey and either a positron emission tomography/computed tomography (PET/CT) scan or magnetic resonance imaging (MRI) of the entire spine and pelvis (2, 6, 7). The treatment of choice for EP is surgery and radiation therapy (RT) with dose of 40 to 50 Gy over a four-week period, the disease is highly radiosensitive. Small lesions may be cured with surgery alone and no adjuvant RT is indicated unless a residual local disease is suspicion (8). In this article, the 2004 to 2010 pathology records of Khalili Hospital, affiliated to Shiraz University of Medical Sciences, Shiraz, Iran, were reviewed and 3 cases of EMP in the nasal cavity were described.

## 2. Case Presentation

### 2.1. Case 1

**Figure 1.**
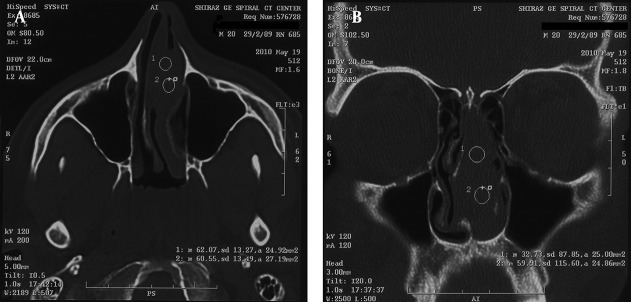
(A, B). CT Scan-Axial View, Left Nasal Cavity Mass with Septal Deviation

**Figure 2.**
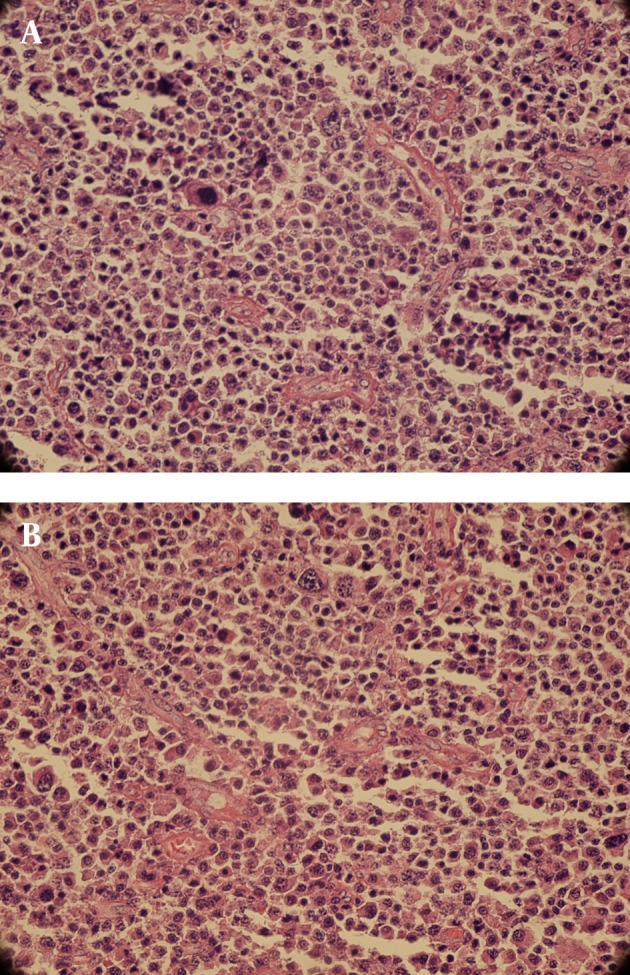
(A, B). Sheet of Plasma Cells with Occasional Giant Binucleated Cells The amorphous amyloid like substance is precipitated around blood vessels and

This case was diagnosed by chance and treated initially with radiation therapy and later with surgery. He is 20 year old with no significant past medical history. The patient was completely asymptomatic and following a paroxysm of sneezing, a small brown color mass was expectorated from nose. CT scan revealed a mass in the left nasal cavity with septal deviation to the right side without bony destruction (Figure 1A, 1B). The nasal mass was completely removed via an endonasal endoscopic approach. Microscopically, the mass consist of a pure population of plasma cells with small, round, and eccentric nucleus, abundant basophilic cytoplasm and perinuclear halo. Few binucleated or multinucleated tumor cells are identified (Figure 2A, 2B). The immunohistochemical study was confirmed plasmacytoma. Bone marrow biopsy was normocellular marrow and laboratory tests did not show anemia, hypercalcemia, or renal failure. No evidence of recurrence following completion of treatment has been detected during one year clinical surveillance.

### 2.2. Case 2

**Figure 3.**
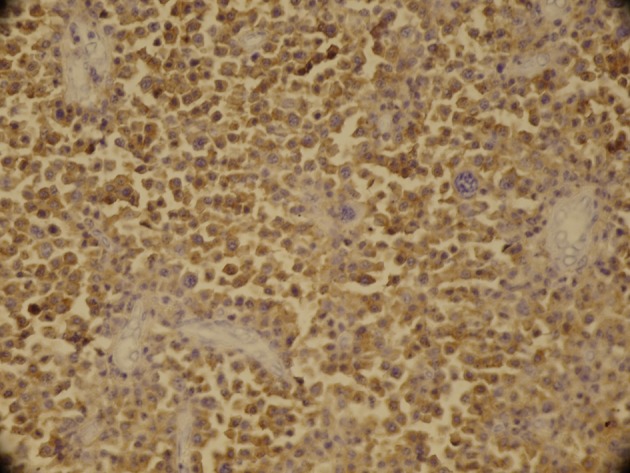
Immunohistochemical Studies with Cd38 Antibody Stain Showed Immunopositivity of Tumor Cells (× 200)

A 48-year-old male presented to our clinic with history of epistaxis. Nasal endoscopy revealed a soft, friable, polypoid mass occupying the left nasal cavity. CT scan of the nasal cavities and paranasal sinuses revealed a mass involving the left medial canthum and the base of the nasal pyramid. Tissue biopsy revealed sheet of discohesive neoplastic plasma cells with eccentric hyperchromatic nuclei with irregular chromatin distribution (Figure 3). The immunohistochemical study was also consistent with plasmacytoma. Results of serum electrophoresis and urine tests were negative for myeloma component or Bence-Jones protein. Bone marrow needle biopsy and skeletal survey were negative. The intranasal mass was totally removed endoscopically with no complications. The patient received postoperative radiotherapy (4400 cGy over a 1-month period). At one year follow-up, the patient was doing well with no signs of recurrence.

### 2.3. Case 3

A 60-year-old male complained of recurrent right-sided epistaxis for three months. Examination under anesthesia revealed a friable and bloody tumor mass at the right middle meatus. Biopsy was taken for histopathological examination revealed sheet of plasma cells. The impression of plasma cell tumor was confirmed by immunohistochemistry on 2008. Urine assays for Bence Jones protein was negative and there was no evidence of gammopathy in serum electrophoresis. A bone marrow biopsy did not demonstrate any skeletal involvement. Bone marrow examination was normal. The patient received radiotherapy and was asymptomatic for more than three year. After three years, the patient admitted due to local recurrence of tumor. Immunohistochemical study was carried out for all patients. Tumor cells were positive for CD38 (Figure 4), EMA and negative for LCA, CD3, CD20 and CK (Dako, Denmark).

## 3. Discussion

Plasmacytoma is a rare solitary mass of neoplastic monoclonal plasma cells, first described by Schridde in 1905 (5). Extramedullary plasmacytoma originate from plasma cells with a single class of heavy and light chains in a monoclonal proliferation of B cells. The commonest immunoglobulin expressed by the tumor cells is IgG with kappa chain restriction. Eighty percent of EMPs arise in the soft tissues of the head and neck region. The nasal cavity, paranasal sinuses, and nasopharynx are the most common sites (9, 10). Extramedullary plasmacytoma represent 3% of plasma cell neoplasms. Tissue biopsy, serum electrophoresis and radiological skeletal survey with bone marrow study are necessary for diagnosis. The treatment of EP is surgical resection and radiotherapy (11). Chemotherapy may be considered for patients with refractory or relapsed disease (8). Follow-up radiological and serum electrophoresis is required after treatment to detect recurrences and progression to myeloma. Approximately 10% of EP has multiple sites of involvement (1). The rate of progression to multiple myeloma is lower than in solitary bone plasmacytoma (SBP), ranging from 11% to 30% and is associated with a poorer prognosis (3). In pathology, plasma cells are present with a morphologic spectrum ranging from mature forms with abundant cytoplasm and perinuclear halo to highly atypical cells with large nuclei, hyperchromatic clumped chromatin, and prominent nucleoli with scant cytoplasm. Amyloid deposition may be seen in 15%–38% of extramedullary plasmacytoma. Immunohistochemistry is used to establish clonality (2, 12). Wiltshaw classified soft-tissue plasmacytoma into 3 clinical stages, as follows: Stage I – Limited to an extramedullary site, Stage II – Involvement of regional lymph nodes and Stage III – Multiple metastasis (11). The 10-year survival rate is 70%. Plasmacytoma should be differentiated from other lesions having plasma cells such as plasma cell granuloma, chronic granulomatous inflammation and rhinoscleroma. Plasma cell granuloma shows an admixture of inflammatory cells including plasma cells, histiocytes, eosinophils and fibroblasts. A chronic granulomatous inflammation is characterized by the presence of epithelioid histiocyte, granulomas and giant cells. Lesions with monoclonal plasma cells are considered neoplastic, whereas lesions with multiclonal plasma cells are inflammatory nature (5).
